# Development and validation of a high-throughput agglutination assay for O-serogrouping clinical *Escherichia coli* isolates

**DOI:** 10.1128/spectrum.00991-26

**Published:** 2026-08-21

**Authors:** Ritu Shastry, Wenchi Shang, Sarah Janssen, Sarah Tete, Elizabeth Garcia Dominguez, Germie van den Dobbelsteen, Todd Davies, Brian Morrow

**Affiliations:** 1 Bacterial Vaccines Discovery and Early Development, Johnson & Johnson Innovative Medicine 6808 , Raritan, New Jersey, USA; 2 Discovery and Manufacturing Statistics, Johnson & Johnson Innovative Medicine 72478 , Archimedesweg, Leiden, the Netherlands; 3 Bacterial Vaccines Discovery and Early Development, Johnson & Johnson Innovative Medicine 502036 , Archimedesweg, Leiden, the Netherlands; University of Georgia College of Veterinary Medicine, Athens, Georgia, USA

**Keywords:** high-throughput O-serotyping, *Escherichia coli*, clinical validation

## Abstract

Accurate O-serotyping of *Escherichia coli* remains essential for clinical surveillance, outbreak investigation, and evaluation of vaccines targeting invasive *E. coli* disease. Traditional agglutination-based serotyping is labor-intensive, low-throughput, and reliant on subjective visual interpretation. We developed a high-throughput (HTP) 96-well agglutination assay incorporating automated OD normalization (BioTek Epoch) and objective agglutination quantification using a CTL ImmunoSpot reader. A data-driven positivity cutoff (Total Intensity of Spots per Well < 11.65) was established via Youden’s index to optimize diagnostic discrimination. Validation was performed in a GCLP-compliant laboratory using clinical isolates representing the nine O-serogroups included in the ExPEC9V vaccine candidate. The assay demonstrated 100% sensitivity across all homologous O-serogroups (*n* = 9) and ≥95% specificity across heterologous O-serogroups, including five non-ExPEC9V clinical isolates. Robustness was confirmed for bacterial suspensions with OD_600_ values of 0.6–1.2, the range recommended for clinical implementation. Long-term stability of boiled cultures stored at 2–8°C was inconsistent—particularly for O75 antisera—supporting same-day preparation of suspensions. Due to heat-associated interference observed with deep-well plastic blocks, glass 96-well tube systems were validated and adopted for clinical testing, demonstrating 100% specificity across all heterologous O-serogroups. This validated HTP agglutination assay provides a scalable, reproducible, and clinically actionable method for *E. coli* O-serotyping. By preserving the immunologic specificity of conventional agglutination while enabling automation and objective readouts, the assay is well suited for vaccine efficacy studies, reference laboratory workflows, and clinical microbiology programs requiring accurate phenotypic confirmation of O-antigen expression.

**IMPORTANCE:**

Agglutination serotyping of bacterial isolates is labor-intensive, requires specialized laboratories and skilled personnel, and relies on subjective visual interpretation. Nevertheless, it remains essential for determining O-antigen expression, providing information that molecular methods—focused only on detecting serotype-specific genes—cannot. For this reason, agglutination O-serotyping continues to serve as the regulatory accepted gold standard. This study describes the development, optimization, and validation of a high-throughput agglutination O-serotyping assay designed to overcome the constraints of low throughput and operator-dependent variability. The validated assay demonstrated its scientific utility through the characterization of clinical isolates from a Phase 3 trial of a candidate *Escherichia coli* vaccine.

**CLINICAL TRIALS:**

This study is registered with ClinicalTrials.gov as NCT04899336.

## INTRODUCTION

Despite its ubiquity as a laboratory organism and a human commensal, *Escherichia coli* includes isolates that are the causative agents of serious, invasive human diseases, including bacteremia, (uro)sepsis, and meningitis (1). Recent epidemiology studies have demonstrated an increasing incidence of invasive *E. coli* disease (IED) (bacteremia and sepsis), particularly among older adults, which is associated with a high fatality rate (2). The increasing incidence of infections is accompanied by a parallel increase in antimicrobial resistance, with drug-resistant *E. coli* accounting for almost 220,000 deaths globally in 2019, and third-generation cephalosporin- and fluoroquinolone-resistant *E. coli* isolates causing more than half of those deaths (3).

The paucity of new agents effective against drug-resistant *E. coli* underscores the importance of identifying alternative means for the prevention or treatment of serious, invasive *E. coli* infections. At present, there is no vaccine available to prevent IED, and ExPEC9V was advanced as a candidate vaccine to prevent invasive infections, including bacteremia. ExPEC9V is comprised of the O-antigens for the most prevalent ExPEC O-serogroups associated with bloodstream infections, with each O-antigen individually bioconjugated to the carrier protein, genetically detoxified *Pseudomonas aeruginosa* exotoxin A (4). The nine O-antigens were selected based on a large global surveillance study of isolates from patients ≥60 years of age hospitalized in Europe, North America, Asia-Pacific, and South America (5) and a large multinational retrospective observational study in IED patients predominantly >60 years of age (6). In both studies, O25 was the most prevalent O-serogroup with O2, O6, O1, O75, O15, O8, O16, O4, and O18 in common among the predominant O-serogroups. Isolates from these targeted O-serogroups not only cause the majority of bloodstream infections, but also cause antimicrobial-resistant IED, and include the multi-drug-resistant virulent clone ST131 (5). A 10-valent candidate vaccine targeting 10 O-serogroups was assessed in a Phase 1/2a study (7), and a nine-valent version was advanced into a Phase 3 clinical study (ClinTrials.gov NCT04899336) assessing the efficacy of ExPEC9V in preventing IED caused by these nine O-serogroups, although this study was terminated due to a lack of efficacy.

The *E. coli* cell envelope is comprised of a lipid A membrane anchor, proximal core polysaccharide, and a distal O-antigen polysaccharide. The surface-exposed O-antigen is comprised of unique polysaccharide structures which differ in the number, order, and linkages of specific sugars, which provides immunological specificity between antigenically unique O-antigens (8), of which there are more than 180 (9). The antigenic classification of *E. coli* by O-serotyping relies on the “gold standard” method, which involves the agglutination of boiled *E. coli* culture preparations by antisera raised against each O-antigen and was originally developed in the 1940s (10). As the standard agglutination method for O-serotyping is tedious, labor-intensive, and has several shortcomings, many laboratories are currently emphasizing molecular methods of typing (11), although DNA sequence analysis also has limitations with regards to typing strains. There is a clear need to develop a high-throughput assay for the characterization of O-serogroups to accommodate a large number of isolates and to incorporate automation, where possible, to reduce the need for hands-on steps. Additionally, as the output from the agglutination assay is historically based on visual assessment by trained operators, the implementation of an automated plate reader would eliminate subjectivity in the interpretation of agglutination reactions that may vary from clearly positive or negative to weakly positive, with agglutination reactions that are neither fully positive nor negative. This would provide for improved consistency both within and between laboratories.

Immunomagnetic separation, in which paramagnetic beads coated with polyclonal antibodies, is commercially available and used for the detection and isolation of Shiga toxin-producing *E. coli* (STEC) typically from contaminated food, although the available beads are limited to those O-serogroups associated with STEC, and these have successfully been used for enrichment from contaminated sources but not for the purpose of O-serotyping (12).

To address the need for a validated, accurate, and reliable approach to O-serotyping *Escherichia coli* isolates, we developed, optimized, and validated a high-throughput agglutination assay for the nine most prevalent O-serogroups associated with *E. coli* bloodstream infections and targeted by the candidate ExPEC9V vaccine. This assay preserves the immunologic specificity of the agglutination “gold standard” while significantly enhancing throughput and operational reliability. The objective of this study was to establish a robust, GCLP-compliant high-throughput agglutination method for O-serotyping *E. coli* isolates, intended for use as a primary endpoint assay to assess the efficacy of a novel vaccine candidate in Phase 3 clinical trials.

## MATERIALS AND METHODS

This paper describes the validation of a high-throughput (HTP) agglutination assay using a 96-well format and automated plate reading to determine the O-serogroups of clinical *E. coli* isolates expressing O-antigens targeted by the ExPEC9V candidate vaccine (O1, O2, O4, O6, O15, O16, O18, O25, and O75). A detailed SOP for this assay is available in Supplemental Materials. Sensitivity and specificity, robustness, and stability parameters were validated in this study.

The assay principle is summarized in Fig. 1. The assay was validated according to available regulatory guidelines (13–16). The agglutination method used was adapted from the procedures recommended by SSI Diagnostica (SSID, Denmark) (17).

**Fig 1:**
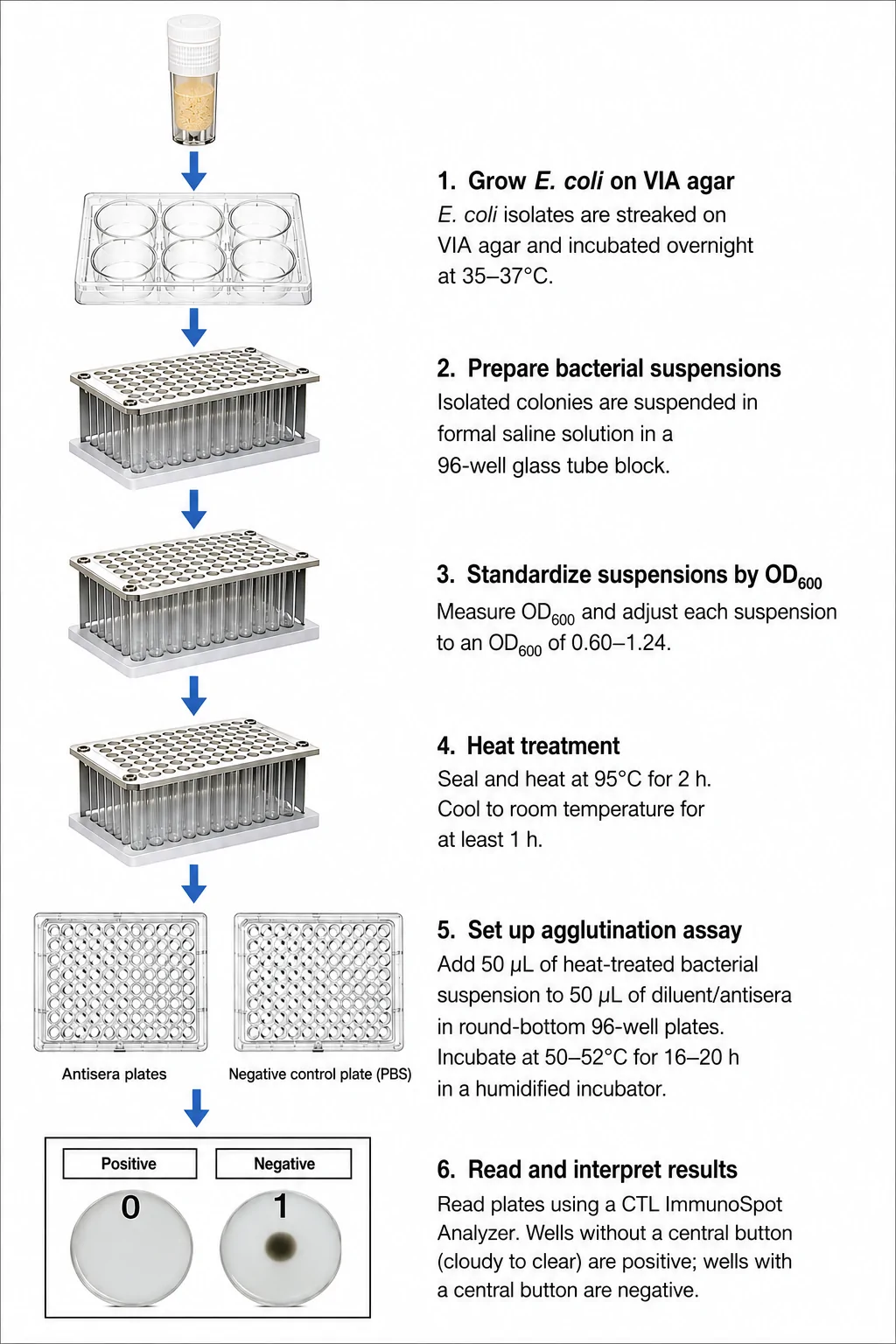
Schematic representation of HTP agglutination assay for O-serogroup typing of *E. coli* isolates. Examples of positive and negative agglutination reactions to O-antisera are illustrated in the example image of two wells from a plate.

*E. coli* from frozen glycerol stocks were incubated overnight at 37°C on Veal Infusion/Yeast extract agar plates prepared in six-well plates (VWR). On the following day, using wide-orifice Rainin tips, isolated colonies were suspended in vented aluminum 96-well glass tubes (Chrom Tech, Inc.) or in a 96-deep well block (Biotix) containing 2 mL of formal saline solution (FSS), which comprises a final concentration of 0.216% formaldehyde and 0.55% NaCl (Millipore Sigma). The colonies were dislodged using a multichannel pipette, followed by thorough mixing of the bacterial suspensions with a 1,250 µL EClip-Tip multichannel pipette (ThermoScientific) to ensure complete resuspension. The optical density at 600 nm (OD_600_) of each well was measured using a BioTek Epoch plate reader with Gen5 software, after transferring 200 µL of the bacterial resuspension into a 96-well Nunc plate (VWR), to achieve a final OD_600_ ranging from 0.6 to 1.24 in each well. Subsequently, the vented aluminum 96-well glass tubes were sealed with a sealing mat and aluminum lid, heated in a 95°C water bath for 2 h, and allowed to cool to ambient temperature for at least 1 h prior to the assay setup.

For the assay setup, high-titer O-antiserum purchased from SSID was diluted with 0.85% saline solution at pH 7.4 (Millipore Sigma) on the day of the assay, adhering to the manufacturer’s instructions. In a 96-well round bottom plate, 50 µL of the boiled and cooled bacterial culture was combined with either 50 µL of diluted O-antiserum or 50 µL of 1× PBS (as a negative control). A positive reaction is characterized by a “cloudy to clear well without a button,” whereas a negative result is indicated by the presence of a small button at the center of the well, as illustrated in Fig. 1. The button located at the bottom of each well, including positive controls, is quantified as the Total Intensity of Spots per well, which was automatically captured by the CTL ELISpot Reader during data acquisition on the CTL ImmunoSpot instrument. At each step of analysis in CTL software (scan and count), plate data were “checked into” the chosen plate directory, preventing files from being modified or deleted using Windows file and folder permissions. This was accomplished by enabling EX Compliance on the CTL Analyzer computers, which allowed us to maintain compliance in a regulated environment. All equipment and software used in the validation study were FDA 21 CFR 11 and GCLP compliant and validated prior to performing the validation.

Results for an individual agglutination test were accepted if the corresponding negative control well (boiled *E. coli* culture mixed with 1× PBS) produced a negative result consisting of a small button centered in the bottom of the well with a Total Intensity of Spots meeting the established cutoff threshold criteria.

The typing antisera typically recognizes the O-antigen from related sub-serogroups (e.g., O25a and O25b), and as such, this HTP agglutination assay did not characterize isolates to the sub-serogroup level. For O18, typing antisera recognizing the O18ac antigen was used in this assay, although in our results, almost all O18 strains were positive for agglutination by both O18ab and O18ac antisera (results not shown).

### Automation of agglutination assay plate reading using the CTL ELISpot Reader and establishment of cutoff thresholds for positive and negative reactions

In our experience during assay development and optimization, it was not uncommon for strains of a confirmed O-serogroup to exhibit slight variations in reactivity to antisera, which may lead to incomplete agglutination. As illustrated in Fig. 1, a spot count of 0 indicates positive agglutination, while a spot count of 1 indicates negative agglutination. Consequently, a very faint button at the bottom of the well could yield either a score of 0 or 1, depending on the intensity of the spot, potentially resulting in false-negative or false-positive agglutination outcomes.

To address this variability, a study was conducted to establish an improved threshold (cutoff value) for reporting positive and negative results. Since the cutoff value is not anticipated to be O-serogroup-dependent, a single cutoff value was calculated across all tested O-serogroups.

To evaluate a potential cutoff value for distinguishing between positive and negative samples, we analyzed total intensity data of spots per well from various assay optimization experiments. These assay runs utilized varying ratios of boiled bacterial suspensions to antisera in 96-deepwell plastic blocks (Biotix). The total intensity of spots per well (%max), referred to as densities, was recorded for *E. coli* isolates using O-antisera from ExPEC 9V O-serogroups and PBS. Isolates measured with homologous O-antisera were classified as true positives, while those with PBS were considered true negatives.

The optimal cutoff was determined through maximizing Youden’s index (*J*), which provides a summary measure of a diagnostic test’s performance. Youden’s *J* statistic ranges from 0 to 1, where a value of 0 indicates that the test performs no better than chance, and a value of 1 reflects perfect classification with no false positives or false negatives. The index gives equal weight to sensitivity and specificity. The cutoff that maximizes Youden’s index is considered optimal, as it represents the point where the test best distinguishes between positive and negative cases. All statistical analyses were conducted using R Studio with R version 4.0.5. An overview of the definitions used is provided in Table 1.

**TABLE 1:** Possible outcomes and definitions^*a*^

	Classified by cutoff		
True status isolate	Positive	Negative	Total
Positive	*a* (true positives)	*b* (false negatives)	*a* + *b*
Negative	*c* (false positive)	*d* (true negatives)	*c* + *d*

^*a*^ Sensitivity = true positive rate = *a*/(*a* + *b*); Specificity = true negative rate = *d*/(*c* + *d*).

Youden’s J statistics:


J=sensitivity+specificity−1

$$J=\frac{\text{true positive}}{\text{true positives}+\text{false negatives}}+\frac{\text{true negatives}}{\text{true negatives}+\text{false positives}}-1$$

$$J=\frac{a}{a+b}+\frac{d}{c+d}-1$$

### *E. coli* strains

Ten *E. coli* strains of each 9V O-serogroup were used during the assay validation, and the assay plate layout is presented in Table 2. Additionally, five non-9V strains were also included in the validation study. The 95 strains used for assay validation were derived from multinational epidemiology or surveillance studies conducted to assess O-serogroup prevalence and antibacterial resistance profiles for isolates causing IED. Isolates were collected between 2002 and 2012, with 86 isolates (90.5%) sourced from blood and 87 (91.6%) isolates from patients ≥60 years of age (Table S1). All isolates were characterized by agglutination O-serotyping using standard methodology, and whole-genome sequence analysis was performed on 63 isolates (European Nucleotide Archive Accession PRJEB97262), with a 100% concordance between both methods, noting that the O44 isolate identified by agglutination typed as O17_O44_O73_O77_O106 by DNA sequence analysis, as this method cannot differentiate between these closely related O-serogroups.

**TABLE 2:** *E. coli* strains and assay plate layout used in HTP agglutination validation

	1	2	3	4	5	6	7	8	9	10	11	12
A	O1-1	O1-2	O1-3	O1-4	O1-5	O1-6	O1-7	O1-8	O1-9	O1-10	O75-1	O75-2
B	O2-1	O2-2	O2-3	O2-4	O2-5	O2-6	O2-7	O2-8	O2-9	O2-10	O75-3	O75-4
C	O4-1	O4-2	O4-3	O4-4	O4-5	O4-6	O4-7	O4-8	O4-9	O4-10	O75-5	O75-6
D	O6-1	O6-2	O6-3	O6-4	O6-5	O6-6	O6-7	O6-8	O6-9	O6-10	O75-7	O75-8
E	O15-1	O15-2	O15-3	O15-4	O15-5	O15-6	O15-7	O15-8	O15-9	O15-10	O75-9	O75-10
F	O16-1	O16-2	O16-3	O16-4	O16-5	O16-6	O16-7	O16-8	O16-9	O16-10	O21	O22
G	O18-1	O18-2	O18-3	O18-4	O18-5	O18-6	O18-7	O18-8	O18-9	O18-10	O44	O88
H	O25-1	O25-2	O25-3	O25-4	O25-5	O25-6	O25-7	O25-8	O25-9	O25-10	O153	FSS

According to SSID (17), the boiled *E. coli* suspensions can be stored at 2–8°C for 1 week but must be mixed again by turning the tubes containing the bacterial suspensions upside down a few times and left for 1 h on the table before use. For the validation study, boiled bacterial suspensions were used on the day of preparation.

### O-antisera

All nine O-antisera were manufactured by SSID and purchased for use in the validation of the assay. These O-antisera were stored at 4°C in a Good Clinical Laboratory Practice (GCLP)-certified and temperature-monitored refrigerator to ensure stability. Unopened vials are stable for 4 years from the date of manufacture (17).

On the day of the assay, a vial of high-titer antisera was opened and diluted following the manufacturer’s instructions. Once diluted, the antisera were only used on the day of the assay and not subsequently. The antisera purchased from SSID included O1, O2, O4, O6, O15, O16, O18, O25, and O75.

### Assay validation parameters

#### Sensitivity and specificity

Sensitivity and specificity were evaluated by testing *E. coli* isolates of known O-serogroups using both homologous and heterologous O-antisera within the same experiment. Homologous antisera are serogroup-specific antisera tested against *E. coli* isolates of the same O-serogroup, such as O1 antisera tested with O1 isolates. Heterologous antisera are tested against *E. coli* isolates of different O-serogroups, such as O2 antisera tested with O6 or O25 isolates.

Each run involved testing ten isolates of each homologous O-serogroup alongside 85 heterologous isolates, which included five other heterologous strains (non-ExPEC9V strains): O21, O22, O44, O88, and O153. They were selected as representatives of non-9V O-serogroups that are frequently encountered in contemporary clinical isolates (18).

In each experimental run, a total of 10 plates were used. One plate served as a negative control, containing 1× PBS, while the remaining nine plates were tested with antisera specific to O1, O2, O4, O6, O15, O16, O18ac, O25, and O75, respectively. Across six valid runs conducted on different days by four qualified operators, we generated a total of 60 data points for each homologous O-serogroup and 510 data points for the heterologous O-serogroups (including the non-9V O-serogroups). An example of the plate layout is presented in Table 2.

Both sensitivity and specificity were evaluated per O-serogroup. The acceptance criteria for sensitivity were claimed if the true positive rate was ≥95% based on the homologous serogroup. The acceptance criteria for specificity were claimed if the true negative rate was ≥95% across all heterologous serogroups, and if the true negative rate was ≥95% per heterologous serogroup with *N* ≥ 20 available results, and if there was a maximum of 1 positive result per heterologous serogroup with *N* < 20 available results.

#### Robustness

The assay’s robustness was assessed using two distinct *E. coli* suspensions: one at a lower optical density (OD) range (OD_600_ = 0.5–0.6) and another at an upper range (OD_600_ = 1.1–1.2). This evaluation aimed to determine the useful range of bacterial suspension ODs for this assay.

Given the labor-intensive requirement of adjusting individual sample OD_600_ levels to a specific range, one representative isolate was selected per O-serogroup in a 96-format. For example, row A contained 10 duplicates of one strain of O1 serogroup occupying wells A1–A10, while rows B–H contained 10 duplicates of one strain of O2, O4, O6, O15, O16, O18, and O25, occupying wells B1–B10, C1–C10, D1–D10, E1–E10, F1–F10, G1–G10, and H1–H10, respectively. Wells A1–A12, B11–B12, C11–C12, D11–D12, and E11–E12 contained 10 duplicates of an O75 strain.

Within each run, 10 replicates of the same isolate for each O-serogroup were tested. Robustness was evaluated across two runs for each robustness condition, resulting in a total of 20 data points for each homologous O-serogroup and 160 data points for the heterologous O-serogroups per robustness condition.

Robustness was evaluated by assessing sensitivity and specificity per O-serogroup using 10 duplicates of a strain representative of each of the nine O-serogroups. Sensitivity was claimed if the true positive rate was ≥95% based on the homologous O-serogroup. Specificity was claimed if the true negative rate was ≥95% per heterologous O-serogroup.

### Stability of boiled *E. coli* cultures for 6 weeks at 2–8°C

Sample stability refers to the ability of the sample to remain unaffected by varying conditions that mimic handling during routine clinical testing. The bacterial suspension was prepared on the day of the assay, and we assessed its potential for storage at 2–8°C for up to 6x weeks for future use.

Stability samples were organized following the same plate layout as used in specificity and sensitivity runs 1 and 2 and were stored for 6 weeks before subsequent testing. The stability of *E. coli* was analyzed across two runs, resulting in 20 data points for each homologous O-serogroup and 160 data points for the heterologous O-serogroups. Non-9V strains were not considered for stability analysis. According to the assay acceptance criteria, individual wells must pass their negative controls (1×× PBS) to qualify for analysis.

To assess stability of boiled cultures at 4°C for 6 weeks, sensitivity and specificity were evaluated per O-serogroup. Sensitivity was claimed if the true positive rate was ≥95% based on the homologous O-serogroup, and specificity was claimed if the true negative rate was ≥95% per heterologous O-serogroup.

### Validation of 96-well glass tubes

During validation of the high-throughput assay using 96-deepwell plastic blocks, we observed inconsistent results characterized by variable button intensities. A subsequent risk assessment indicated that these discrepancies were likely caused by interference from plastic material during agglutination reactions after heating at 95°C. Because the original method used glass tubes (17) and given the potential for material-related assay interference, it was necessary to identify a suitable alternative in the event of plastic block unavailability or incompatibility.

We therefore conducted a new validation using glass tubes to assess sensitivity and specificity, while retaining the previously validated robustness OD conditions. Vented aluminum 96-well plate bases fitted with 2.5 mL insertable glass tubes (ChromTech, Inc.) were selected as the replacement format. This configuration proved suitable for the HTP agglutination assay and was used for Phase 3 clinical testing.

The validation of the high-throughput agglutination assay was conducted using 96-deepwell plastic blocks, during which we evaluated sensitivity, specificity, robustness, and stability. In contrast, the assessment of glass tubes focused solely on sensitivity and specificity. A total of four runs were performed by testing *E. coli* isolates of known O-serogroups with homologous and heterologous O-antisera in the same experiment. Ten isolates of each homologous O-serogroup were tested together with 80 heterologous isolates in each run. Each run comprised 10 plates, with each plate tested with O-antisera or 1× PBS (negative control), and generated a total of 40 data points per homologous O-serogroup and 320 data points for the heterologous O-serogroups.

## RESULTS

### Cutoff determination

#### Data exploration

Three ratios of bacterial suspensions to antisera were tested for agglutination with homologous and heterologous typing antisera for all reference strains, with the density values plotted in Fig. 2. In this figure, the highest density values were observed for PBS, with the lowest density values observed for positive agglutination reactions for each tested antisera and strains expressing the corresponding O-antigen. These density values permitted the determination of a statistically determined cutoff value differentiating positive and negative agglutination reactions. Figure 3 illustrates the density plots of the density readout per condition, highlighting the true status of the isolates. PBS are high-density values in pink, other serogroups in different colors are low on density as seen on the *Y*-axis. The densities depend on the condition.

**Fig 2:**
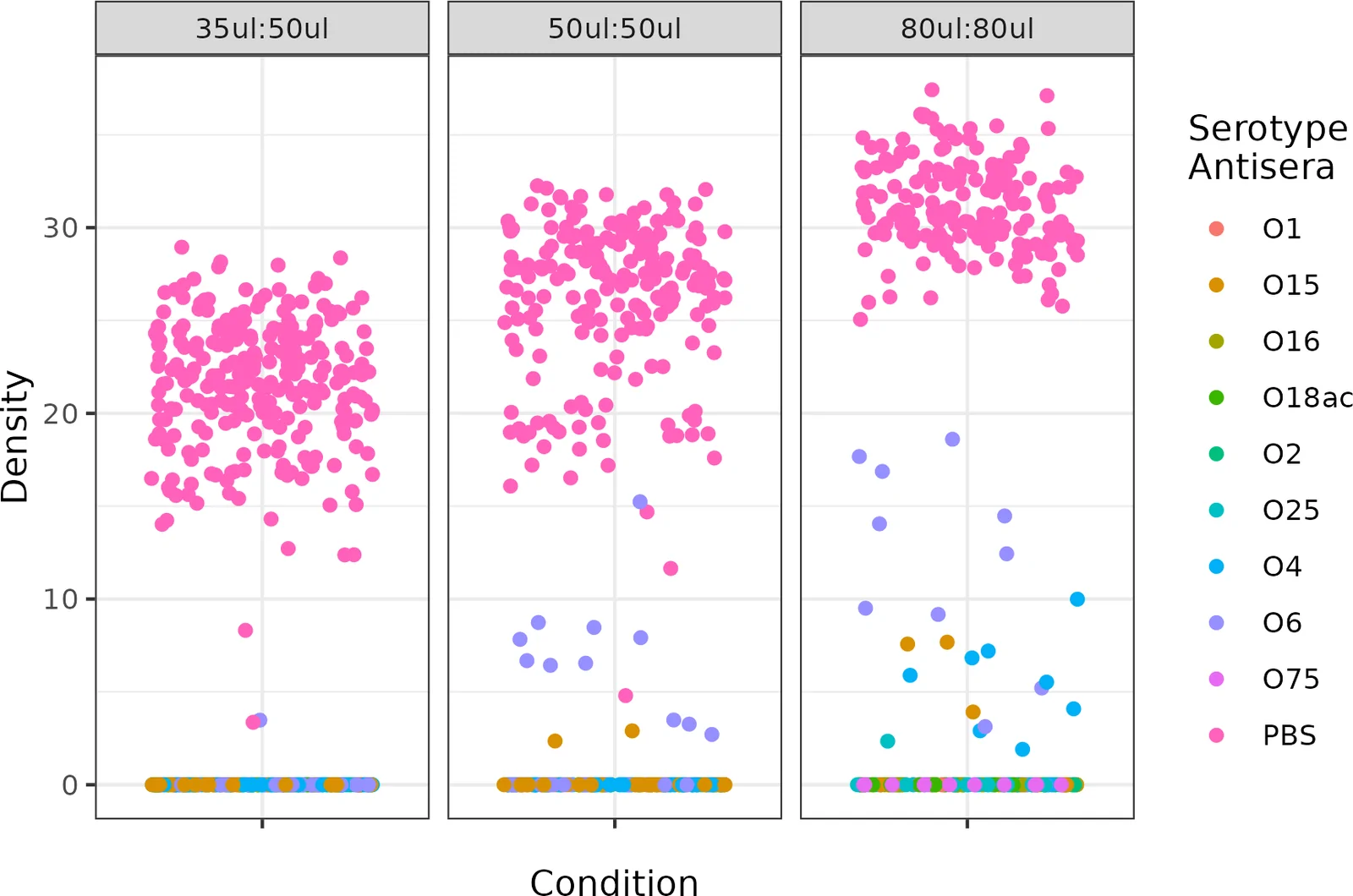
Density (*Y*-axis) versus condition (*X*-axis, Panels). Three ratios of bacterial suspensions to antisera, 35 µL:50 µL, 50 µL:50 µL, and 80 µL:80 µL, were tested, with density values plotted with different colors representing either PBS or specific typing antisera. Negative agglutination reactions with PBS were observed as high-density values, with positive reactions generating low-density values at the bottom of each plot.

**Fig 3:**
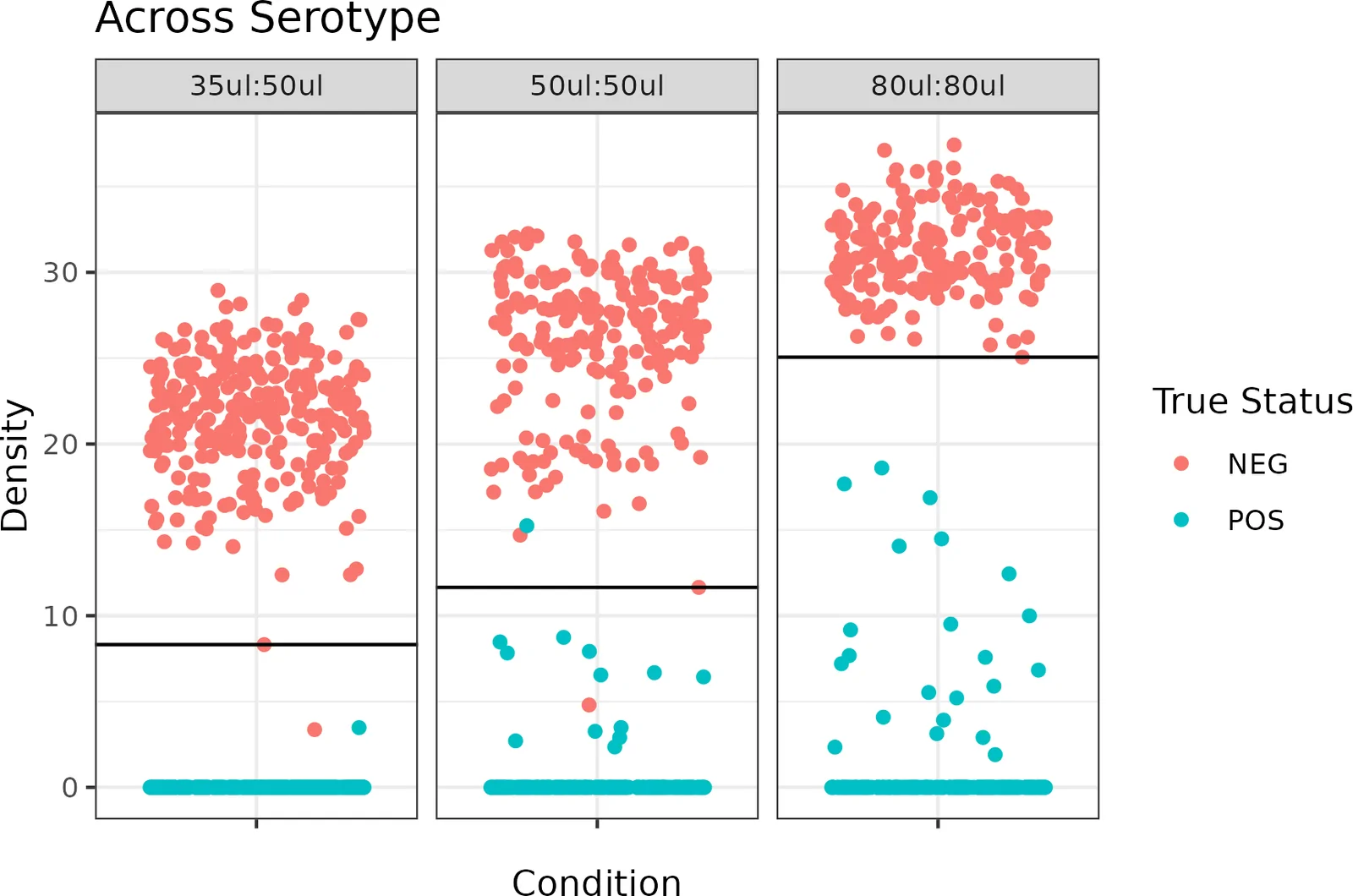
Density (*Y*-axis) versus condition (*X*-axis/Panels). The red and blue colors refer to the true negative and true positive, respectively. The black horizontal line refers to the optimal cutoff.

#### Statistical determination of cutoff threshold for agglutination values

The optimal cutoff values for the three ratios of boiled bacterial suspensions to antisera—35 µL:50 µL, 50 µL:50 µL, and 80 µL:80 µL—are presented in Table 3 and visually represented in Fig. 3. Table 3 details the total counts of negatives and positives, alongside the number of false positive and false negative results, as well as their respective percentages for each condition.

**TABLE 3:** Optimal cutoff per condition

Condition	Optimal Cutoff	No. of negative	No. of false positive	False positive (%)	No. of positive	No. of false negative	False negative (%)
35 µL:50 µL	8.31	275	2	0.7	260	0	0.0
50 µL:50 µL	11.65	210	1	0.5	200	1	0.5
80 µL:80 µL	25.06	190	0	0.0	180	0	0.0

Among these, an optimal cutoff of 11.65 was established using the bacterial suspension to antisera ratio of 50 µL:50 µL. This selection was made to minimize the number of antisera vials purchased while ensuring adequate volume of bacterial suspension for conducting the assay. A Total Intensity of Spots per Well (%max, as calculated using the CTL software) of ≥11.65 indicated a negative result, whereas a value of <11.65 indicated a positive result.

### Sensitivity and specificity with 96-deepwell plastic blocks

In validation studies conducted with 96-deepwell plastic blocks, the sensitivity acceptance criteria of true positive rates of ≥95% were met for all homologous serogroups. The observed true positive rates were 100% for all nine serogroup strains as all 60 data points per homologous serogroup passed. The sensitivity results for all serogroups are provided in Supplemental Materials.

Specificity was assessed across all heterologous O-serogroups and per heterologous O-serogroup.

In validation studies conducted with 96-deepwell plastic blocks, the specificity acceptance criteria of true negative rates of ≥95% were met for assessment across all heterologous O-serogroups (*n* = 510) with a true negative rate of 100% being observed for all O-antisera except for O75, which displayed a true negative rate of 99.6%.

For each of the EXPEC9V heterologous serogroups, specificity was 96.7–100% meeting the pre-defined acceptance criteria of ≥95% true negative rate. For the O18 heterologous O-serogroup when tested with O75 antisera, 58/60 data points passed at 96.7% still fulfilling the acceptance criteria of ≥95%.

Additionally, the tested strains expressing the non-ExPEC9V O-serogroups O21, O22, O44, O88, and O153 met the acceptance criteria for having a maximum of 1 positive result per heterologous O-serogroup with *N* < 20. The specificity results for all O-serogroups are provided in the Supplemental Materials.

### Robustness

To assess robustness, sensitivity and specificity were evaluated per O-serogroup and per robustness condition using 96-deepwell plastic blocks only. Sensitivity results for all O-antisera are provided in the Supplemental Materials.

For O1, O2, O4, O6, O15, O16, O18, and O25 robustness, specificity acceptance criteria were met across all O-serogroups and with each heterologous O-serogroup tested, with a specificity of 95–100%, meeting the pre-defined acceptance criteria of a ≥95% true negative rate.

For O75 robustness, a true negative rate of 100% was observed for high OD_600_ (1.1–1.2) with all 20 data points passing for all O-serogroups, meeting the pre-defined acceptance criteria of ≥95%. Robustness for O75 O-antisera at low OD_600_ was 85% for serogroup O4, with 17/20 data points passing; 0% for serogroup O18, with 0/20 data points passing; and 70% for serogroup O25, with 14/20 data points passing; therefore, not meeting the pre-defined acceptance criteria of ≥95%.

Therefore, the assay was considered robust at high OD_600_ (1.1–1.2) for all O-serogroups. Robustness at low OD was not demonstrated for O75 anti-sera. As the *E. coli* strains to be tested in clinical studies will be of an unknown serogroup, the low OD_600_ (0.5–0.6) will not be used. Bacterial suspensions will be prepared at the acceptable OD_600_ 0.6–1.2.

### Stability of *E. coli* cultures

Stability of *E. coli* cultures was only assessed with 96-deepwell plastic blocks. To assess stability of boiled cultures at 4°C for 6 weeks, sensitivity and specificity were evaluated per O-serogroup in a subset of samples.

Sensitivity criteria were met for all O-antisera across all nine O-serogroups with true positive rates of 100% for all nine homologous O-serogroups.

Specificity acceptance criteria for 6 weeks were met for all O-serogroups except for O75 O-antisera, which was at 90% when tested with O-serogroup O18, not meeting the pre-defined acceptance criteria of ≥95%.

Since not all the bacterial strain suspensions were stable for 6 weeks, it was decided to prepare fresh bacterial suspensions on the assay day. If a retest is needed, a new suspension will be prepared fresh.

### Sensitivity and specificity with 96-well glass tubes

In validation studies conducted with 96-deepwell plastic blocks, the sensitivity acceptance criteria of true positive rates of ≥95% were met for all homologous serogroups. The observed true positive rates were 100% for all nine serogroup strains, as all 60 data points per homologous serogroup passed. Similar results were also observed during validation of the assay with 96-well glass tubes, where all 40 data points per homologous serogroup passed. The sensitivity results for all serogroups are provided in Supplemental Materials.

Specificity was assessed across all heterologous O-serogroups and per heterologous O-serogroup.

In validation studies using 96-well glass tubes, acceptance criteria were met for all O-serogroups. For all nine antisera (O1, O2, O4, O6, O15, O16, O18, O25, and O75), specificity acceptance criteria of true negative rates ≥95% were met for assessment across all heterologous O-serogroups (*n* = 320), with a true negative rate of 100% being observed per heterologous O-serogroup. The specificity results for all O-serogroups are provided in the Supplemental Materials.

## DISCUSSION

The antigenic classification of *E. coli* by O-serotyping—the traditional “gold standard” method that relies on the agglutination of boiled culture preparations with antisera against individual O-antigens—dates to the 1940s (10). In parallel, serotyping of the *Streptococcus pneumoniae* capsular polysaccharide targeted by currently licensed vaccines relies on immunologic methods such as the Quellung reaction and latex agglutination, which remain the gold standard for isolate serotyping (19). However, these agglutination-based approaches are tedious, labor-intensive, and have several drawbacks, prompting many laboratories to emphasize molecular typing (11, 20), though DNA sequence analysis does not demonstrate the expression of O-antigens for potential recognition by antibodies. Immunomagnetic separation uses commercially available paramagnetic beads coated with polyclonal antibodies to detect and isolate Shiga toxin-producing *E. coli* (STEC) from contaminated food. These beads target only STEC-associated O-serogroups and have proven effective for enrichment from contaminated sources but not for O-serotyping (12). Although phenotypic characterization of O-antigen or capsule serotyping through immunologic methods remains the gold standard for both *E. coli* and *S. pneumoniae* and is the most widely used method in reference labs, as well as the most widely accepted method by public health organizations, validated high-throughput assays have yet to be established.

Although the capabilities to perform whole-genome sequencing and analysis are absent from some clinical laboratories, this technique is gaining widespread utility, and several tools have been developed to take advantage of molecular methods, including PCR (21) and DNA sequence analysis, to characterize O-serogroups through the analysis of O-serogroup-specific genes. Examples of molecular tools to predict O-serogroups include SerotypeFinder (22) and AgriSeq (11). Comparisons between serologic and molecular typing methods reveal strong correlations between these methodologies, although both methods have limitations. In the development of SerotypeFinder, more than 98% of tested isolates were predicted to carry the serogroup-specific O-antigen processing genes matching the O-serogroup determined through agglutination, although 37 isolates were characterized to carry the serogroup-specific *wzx/y* or *wzm/t* genes from different O-serogroups in the same assembly (22). A global surveillance study of bacteremic *E. coli* blood isolates characterized by whole-genome sequence analysis also identified isolates with matches to multiple O-serogroups for DNA sequences within the same genome assembly (5). DNA sequence analysis is not informative as to which, if any, of these multiple O-antigen gene clusters are expressed as O-antigen. Additionally, a large multinational epidemiology study characterizing IED isolates by both agglutination and DNA sequence analysis demonstrated a difference between the two methods, in that the number of IED isolates per O-serogroup was consistently lower by agglutination than by whole-genome sequencing across O-serogroups, indicating possible overrepresentation by molecular typing (6). As some isolates are nontypeable by either serologic or molecular methods, or both, the limitations of each method may be at least partially compensated for by a robust combination of both phenotypic and genotypic characterization of isolates in critical settings.

To establish a robust, high-throughput, automated method for serologic O-serogrouping *E. coli* isolates, a validated high-throughput agglutination assay is needed. Given that agglutination outputs have historically depended on visual scoring by trained operators, implementing an automated plate reader would reduce subjectivity and improve consistency, reliability, and throughput within and between laboratories.

The purpose of this study was to validate an HTP agglutination assay to determine the O-serogroup of *E. coli* isolates corresponding to the O-serogroups found in the ExPEC9V candidate vaccine, which include O1, O2, O4, O6, O15, O16, O18, O25, and O75. Parameters assessed included specificity, sensitivity, robustness, and stability of boiled *E. coli* cultures at 2–8°C.

In developing this high-throughput agglutination assay for O-serogrouping clinical *E. coli* isolates, the workflow was designed to integrate automated instrumentation to improve assay consistency, throughput, and objectivity. Bacterial suspensions were normalized using a BioTek Epoch plate reader, enabling precise OD_600_ measurements in a 96-well format before heating at 95°C and serologic testing. Agglutination reactions were then quantified using the CTL ImmunoSpot (ELISpot) Reader, which replaced subjective visual scoring with automated capture of Total Intensity of Spots per well, allowing for the establishment of a statistically derived cutoff that distinguished positive from negative reactions across serogroups. This combined design—plate reader-driven standardization of bacterial OD_600_ and ELISpot-reader automated agglutination readout—formed the basis of a fully implemented, GCLP-compliant, high-throughput system capable of reliably serogrouping large panels of *E. coli* isolates for the Phase 3 clinical study. This high-throughput agglutination assay was validated for use in the characterization of clinical isolates and demonstrated sensitivity with true positive rates of 100% for all nine homologous O-serogroup strains, and robustness for bacterial suspensions at high OD_600_. This method was used to characterize O-serogroups in clinical isolates from a Phase 3 study of the candidate vaccine ExPEC9V, although this study was terminated due to a lack of efficacy.

While 96-deepwell plastic blocks were validated as fit-for-purpose, the identification of a suitable alternative in case of material unavailability and interference from plastic material when heated at 95°C required us to explore other options. As the original methodology employed glass tubes (17), we selected the vented aluminum 96-well plate bases equipped with 2.5 mL insertable glass tubes from ChromTech, Inc., which demonstrated suitability for the HTP agglutination assay and was used in Phase 3 clinical testing.

The acceptance criteria using 96-well glass tubes were met for all O-serogroups. For all nine antisera (O1, O2, O4, O6, O15, O16, O18, O25, and O75), specificity acceptance criteria of true negative rates ≥95% were met for assessment across all heterologous O-serogroups (*n* = 320), with a true negative rate of 100% being observed per heterologous O-serogroup.

Limitations of this study include that development and validation of a high-throughput O-serogrouping assay was focused on the nine O-serogroups comprising the candidate ExPEC vaccine, as these were the most prevalent in global surveillance and epidemiology studies of bloodstream isolates. As the diversity of *E. coli* O-antigens is extensive, and additional O-serogroups cause IEDs and other infections, the assay in its current form cannot be used to characterize isolates from non-9V O-serogroups. As typing antisera are commercially available for most of the characterized O-serogroups, these may be used in future studies to potentially expand the scope of this assay, although each would need to be validated for sensitivity, specificity, and robustness. As a single cutoff value was successfully applied to identify isolates across nine O-serogroups, expansion of the assay to include additional O-antigens may be straightforward using the methods described here. The expression of capsular polysaccharide by some *E. coli* isolates of the O8 and O9 serogroups (9) may complicate the preparation of bacterial lysates for isolate typing, as isolates expressing a heat-resistant capsule may require modifications to the preparation of bacterial lysates for their successful identification. Additionally, isolates may be nontypeable by agglutination with typing antibodies due to a lack of expression of the O-antigen or ambiguous agglutination results observed as positive reactions to more than one typing antiserum (5, 11). These caveats are not unique to the current, validated high-throughput assay but are shared by all serologic typing assays.

In conclusion, the HTP agglutination assay was validated with acceptable sensitivity and specificity for O-serogrouping of the nine *E. coli* strains targeted in the ExPEC9V candidate vaccine and is hereby designated as validated and fit-for-purpose. This HTP agglutination method may be readily adapted to the characterization of other O-serogroups, including those associated with environmental contamination and foodborne illnesses, including Shigatoxin-producing *E. coli* or others. Additionally, a HTP serologic typing assay may benefit the development of other vaccines or immunotherapies to prevent bacterial neonatal meningitis, for which *E. coli* is the leading cause (23). The development of typing assays incorporating an automated plate reader may also potentially be adapted to the capsular serotyping of *S. pneumoniae* and *Klebsiella* spp. using latex-agglutination methodologies (24, 25).

## Supplementary Material

Reviewer comments

## SUPPLEMENTAL MATERIAL

Supplemental Material spectrum.00991-26-s0001.docx Strain information and requested protocol.

OPEN PEER REVIEW reviewer-comments.pdf An accounting of the reviewer comments and feedback.

ASM does not own the copyrights to Supplemental Material that may be linked to, or accessed through, an article. The authors have granted ASM a non-exclusive, world-wide license to publish the Supplemental Material files. Please contact the corresponding author directly for reuse.
