# Supplementary material for: Development and validation of a high-throughput agglutination assay for O-serogrouping clinical Escherichia coli isolates
Source: Microbiol Spectr. 2026 Aug 21;14(10):e00991-26. doi: 10.1128/spectrum.00991-26 (PMC13689272; doi:10.1128/spectrum.00991-26)
Supplement: Supplementary file 1 [file reviewer-comments.pdf]

ASM | Microbiology Spectrum

Development and Validation of a High-Throughput Agglutination Assay for O-Serotyping Clinical Escherichia coli Isolates

Ritu Shastry, Wenchi Shang, Sarah Janssen, Sarah Tete, Elizabeth Dominguez, Germie van den Dobbelsteen, Todd Davies, and Brian Morrow

Corresponding Author(s): Germie van den Dobbelsteen, Johnson & Johnson Innovation LLC

Review Timeline:

Submission Date:	March 21, 2026
Editorial Decision:	April 16, 2026
Revision Received:	July 2, 2026
Accepted:	July 27, 2026

Editor: Catherine Logue

Reviewer(s): The reviewers have opted to remain anonymous.

Transaction Report:

(Note: With the exception of the correction of typographical or spelling errors that could be a source of ambiguity, letters and reports are not edited. The original formatting of letters and referee reports may not be reflected in this compilation.)

DOI: https://doi.org/10.1128/spectrum.00991-26

Re: Spectrum00991-26 (Development and Validation of a High-Throughput Agglutination Assay for O-Serotyping Clinical Escherichia coli Isolates)

Dear Dr. Germie P.J.M. van den Dobbelsteen:

Thank you for the privilege of reviewing your work. Below you will find my comments, instructions from the Spectrum editorial office, and the reviewer comments.

Please note both reviewer's have expressed concerns regarding the strains mentioned in this study and if they will be available publicy which would limit further validation or analysis of this study.

Please return the manuscript within 60 days; if you cannot complete the modification within this time period, please contact me. If you do not wish to modify the manuscript and prefer to submit it to another journal, notify me immediately so that the manuscript may be formally withdrawn from consideration by Spectrum.

Revision Guidelines
To submit your modified manuscript, log into the submission site at https://spectrum.msubmit.net/cgi-bin/main.plex. Go to Author Tasks and click the appropriate manuscript title to begin. The information you entered when you first submitted the paper will be displayed; update this as necessary. Note the following requirements:

• Upload point-by-point responses to the issues raised by the reviewers in a file named "Response to Reviewers," NOT in your cover letter.
• Upload a compare copy of the manuscript (without figures) as a "Marked-Up Manuscript" file.
• Upload a clean .DOC/.DOCX version of the revised manuscript and remove the previous version.
• Each figure must be uploaded as a separate, editable, high-resolution file (TIFF or EPS preferred), and any multipanel figures must be assembled into one file.
• Any supplemental material intended for posting by ASM should be uploaded with their legends separate from the main manuscript. You can combine all supplemental material into one file (preferred) or split it into a maximum of 10 files with all associated legends included.

For complete guidelines on revision requirements, see our Submission and Review Process webpage. Submission of a paper that does not conform to guidelines may delay acceptance of your manuscript.

Data availability: ASM policy requires that data be available to the public upon online posting of the article, so please verify all links to sequence records, if present, and make sure that each number retrieves the full record of the data. If a new accession number is not linked or a link is broken, provide Spectrum production staff with the correct URL for the record. If the accession numbers for new data are not publicly accessible before the expected online posting of the article, publication may be delayed; please contact production staff (Spectrum@asmusa.org) immediately with the expected release date.

Publication Fees: For information on publication fees and which article types are subject to charges, visit ourwebsite. If your manuscript is accepted for publication and any fees apply, you will be contacted separately about payment during the production process; please follow the instructions in that e-mail. Arrangements for payment must be made before your article is published.

ASM Membership: Corresponding authors may join or renew ASM membership to obtain discounts on publication fees. Need to upgrade your membership level? Please contact Customer Service at Service@asmusa.org.

The ASM Journals program strives for constant improvement in our submission and publication process. Please tell us how we can improve your experience by taking this quick Author Survey.

Thank you for submitting your paper to Spectrum.

Sincerely,
Catherine Logue
Editor
Microbiology Spectrum

Reviewer #1 (Comments for the Author):

This manuscript by Shastry et al describes an E. coli serotyping platform for identification of nine O groups targeted by the ExPEC9V vaccine developed by Johnson & Johnson. The authors develop a system for automated interpretation of serotyping results, and demonstrate >95% sensitivity and specificity (a criteria they defined as acceptable). They identify limited instances

where the assay falls short (O75 antisera when using lower culture dilutions; lines 356-361; stability of boiled cultures; lines 367-377) and propose logical solutions. The scope statement for Spectrum includes "methodological advances and detailed experimental protocols that are of interest to microbiologists", which this study seems to satisfy, especially for researchers needing to quickly screen for the most common O groups of extraintestinal pathogenic E. coli.

Major comments:

One shortcoming noted: Table 2: the assay was validated using a limited number of strains outside the nine tested serogroups. The authors provide an explanation for selecting these out-groups (lines 213-214) and indeed it would be cumbersome to include all other O groups. Perhaps the authors could run a blinded test using in- and more out-group strains to convince the reader this assay would hold up in a more realistic setting?

The Discussion section, especially lines 434-440, 441-451, 452-468, largely repeat the Results. The Discussion should put your results into perspective of previous literature. Other than the first paragraph, there is no literature cited in the Discussion.

Other comments, the first being most critical:

Lines 95-97: more information is needed about the strains used to develop this assay (where and when isolated, how were O groups validated, etc). Will these strains be available to the larger community for validation (as they should be for a manuscript published in an ASM journal)?

Figure legends should provide enough info that reader doesn't need to refer back to the text; I don't feel this is true for Figure 2

Lines 325-326: methods should not be restated in Results except in limited cases needed for clarification

Reviewer #2 (Comments for the Author):

This manuscript provides a derivation of traditional phenotypic serogroup typing assay into a higher throughput method using 96-well plates and a Biotek reader. The study examined nine common serogroups of E. coli which cause IED in humans and tested multiple strains belonging to these groups in a plate assay targeting those nine serogroups.

Overall the work performed was technically sound, although limited by the number of strains and serogroups tested. I have the following comments for improvement.

The authors need to provide more background on the EXPEC9V vaccine and the rationale for the need for only targeting these nine serotypes. They should also acknowledge the current status of the vaccine development, which includes a ceased phase 3 trial because it was ineffective at protecting against IED.

Given that the vaccine mentioned is currently not in use and phase 3 trials are ceased, the authors should provide more attention to the broader uses of this new tool. It is mentioned in the discussion but very briefly.

More details on the strains used for assay validation should be provided. Strain names, sources of isolation, dates of isolation, disease presentation, etc. Are genome sequences available for these strains? It would be nice to have molecular confirmation of their serogroups, sequence types, and virulence factors.

The statement with regards to molecular serotyping that "DNA sequence analysis is not informative with regards to antigen expression" is misleading and not accurate. There have been multiple studies done on this and accuracy of molecular typing compared to phenotypic assays exceeds 95%. See https://journals.asm.org/doi/10.1128/jcm.00008-15

It is worth noting and discussing that while these may be the top nine serogroups from some recent reports, there is a large array of serogroups causing IED and other extra intestinal disease, so the utility of this assay is limited by serogroups tested and overall strains tested.

The density plots provided could be better explained in the text. It was not clear exactly what the plots were demonstrating.

Since the authors conclude that this assay is "is hereby designated as validated and fit-for-purpose," a detailed finalized protocol would be useful with this manuscript so that others can reproduce the work.

No conflict of interest statements were provided for the study and no sources of funding. This should be included.

I would recommend a change to the title because use of the word serotyping (even O-serotyping) suggests complete O:H:K

typing. This method is really O serogroup typing. This should also be changed throughout the manuscript where the term serotyping is used.

Response to Reviewers

Manuscript Title: Development and Validation of a High-Throughput Agglutination Assay for O-Serogrouping Clinical *Escherichia coli* Isolates

Reviewer #1 (Comments for the Author):

This manuscript by Shastry et al describes an E. coli serotyping platform for identification of nine O groups targeted by the ExPEC9V vaccine developed by Johnson & Johnson. The authors develop a system for automated interpretation of serotyping results, and demonstrate >95% sensitivity and specificity (a criteria they defined as acceptable). They identify limited instances where the assay falls short (O75 antisera when using lower culture dilutions; lines 356-361; stability of boiled cultures; lines 367-377) and propose logical solutions. The scope statement for Spectrum includes "methodological advances and detailed experimental protocols that are of interest to microbiologists", which this study seems to satisfy, especially for researchers needing to quickly screen for the most common O groups of extraintestinal pathogenic E. coli.

Major comments:

Reviewer 1, Comment 1

One shortcoming noted: Table 2: the assay was validated using a limited number of strains outside the nine tested serogroups. The authors provide an explanation for selecting these out-groups (lines 213-214) and indeed it would be cumbersome to include all other O groups. Perhaps the authors could run a blinded test using in- and more out-group strains to convince the reader this assay would hold up in a more realistic setting?

Author response: The surveillance and epidemiology studies underlying the selection of the targeted O-serogroups have been expanded upon in the Introduction. This validated HTP assay was used as a component of a Phase 3 clinical study evaluating the efficacy of the candidate vaccine ExPEC9V, and manuscripts presenting the results of this clinical study are in preparation. This provided real-world validation of this assay. Unfortunately, most of the authors are no longer employed by Johnson & Johnson, and additional experimental studies are not currently possible.

Reviewer 1, Comment 2

The Discussion section, especially lines 434-440, 441-451, 452-468, largely repeat the Results. The Discussion should put your results into perspective of previous literature. Other than the first paragraph, there is no literature cited in the Discussion.

Author response: We agree. The repetition of results have been removed from the Discussion, and the Discussion has been rewritten to put the development of this assay into context with other methodologies.

Other comments, the first being most critical:

Reviewer 1, Comment 3

Lines 95-97: more information is needed about the strains used to develop this assay (where and when isolated, how were O groups validated, etc). Will these strains be available to the larger community for validation (as they should be for a manuscript published in an ASM journal)?

Author response: This is a very relevant comment. Details regarding the composition of the validation strain panel have been added to the Methods section, and a Supplemental table has been created presenting metadata, where available, and the characterization details for each of these isolates. These isolates will be available to the scientific community, and the whole genome sequence assemblies have been uploaded to the European Nucleotide Archive, accession number PRJEB97262.

Reviewer 1, Comment 4

Figure legends should provide enough info that reader doesn't need to refer back to the text; I don't feel this is true for Figure 2

Author response: The text has been modified to more clearly explain the data presented in Figure 2, and the legend to this figure has also been modified to provide better clarity.

Reviewer 1, Comment 5

Lines 325-326: methods should not be restated in Results except in limited cases needed for clarification

Author response: The use of the statistical software package was removed from the Results section and moved to the Methods section.

Reviewer #2 (Comments for the Author):

This manuscript provides a derivation of traditional phenotypic serogroup typing assay into a

higher throughput method using 96-well plates and a Biotek reader. The study examined nine common serogroups of E. coli which cause IED in humans and tested multiple strains belonging to these groups in a plate assay targeting those nine serogroups.

Overall the work performed was technically sound, although limited by the number of strains and serogroups tested. I have the following comments for improvement.

Reviewer 2, Comment 1

The authors need to provide more background on the EXPEC9V vaccine and the rationale for the need for only targeting these nine serotypes. They should also acknowledge the current status of the vaccine development, which includes a ceased phase 3 trial because it was ineffective at protecting against IED.

Author response: Conclusions from the surveillance and epidemiology studies supporting the selection of the O-serogroups targeted by the candidate ExPEC9V vaccine were added to the Introduction. The status of the Phase 3 study assessing efficacy of ExPEC9V has also been added to the text.

Reviewer 2, Comment 2

Given that the vaccine mentioned is currently not in use and phase 3 trials are ceased, the authors should provide more attention to the broader uses of this new tool. It is mentioned in the discussion but very briefly.

Author response: The Discussion has been expanded to include a potentially broader use of the assay in the characterization of *E. coli* isolates from O-serogroups beyond those included in the ExPEC9V candidate vaccine, as well as potential use in the serologic characterization of other bacterial organisms.

Reviewer 2, Comment 3

More details on the strains used for assay validation should be provided. Strain names, sources of isolation, dates of isolation, disease presentation, etc. Are genome sequences available for these strains? It would be nice to have molecular confirmation of their serogroups, sequence types, and virulence factors.

Author response: Details on the origins and characterization of the validation strain panel have been summarized in the Methods section, and a table including available metadata has also been included. A total of 63 of the 95 isolates were characterized by whole genome sequence analysis, with a 100% concordance with the agglutination data. Genome sequence assemblies

have been uploaded to the European Nucleotide Archive, accession number PRJEB97262.

Reviewer 2, Comment 4

The statement with regards to molecular serotyping that "DNA sequence analysis is not informative with regards to antigen expression" is misleading and not accurate. There have been multiple studies done on this and accuracy of molecular typing compared to phenotypic assays exceeds 95%. See https://journals.asm.org/doi/10.1128/jcm.00008-15

Author response: This statement has been modified, and a discussion has been added addressing the development of tools to predict O-serogroups from DNA sequences and their application alongside serologic typing assays. The extent of concordance and the limitations of each assay are also addressed.

Reviewer 2, Comment 5

It is worth noting and discussing that while these may be the top nine serogroups from some recent reports, there is a large array of serogroups causing IED and other extra intestinal disease, so the utility of this assay is limited by serogroups tested and overall strains tested.

Author response: Limitations of this study, including the fact that the assay was developed and optimized only with the most prevalent O-antigens in blood isolates, have been added to the text. Additionally, the potential expansion of this assay to include additional O-serogroups and to potentially characterize other bacterial organisms have also been added to the text.

Reviewer 2, Comment 6

The density plots provided could be better explained in the text. It was not clear exactly what the plots were demonstrating.

Author response: The text has been modified to more clearly explain the data presented in Figure 2, and the legend to this figure has also been modified to provide better clarity.

Reviewer 2, Comment 7

Since the authors conclude that this assay is "is hereby designated as validated and fit-for-purpose," a detailed finalized protocol would be useful with this manuscript so that others can reproduce the work.

Author response: A detailed, finalized protocol has been added as supplemental material.

Reviewer 2, Comment 8

No conflict of interest statements were provided for the study and no sources of funding. This should be included.

Author response: All authors were employees of Johnson & Johnson at the time the study was conducted, and funding was provided by Johnson & Johnson. This has been included in the manuscript. This has been added as a separate section before the cited references.

Reviewer 2, Comment 9

I would recommend a change to the title because use of the word serotyping (even O-serotyping) suggests complete O:H:K typing. This method is really O serogroup typing. This should also be changed throughout the manuscript where the term serotyping is used.

Author response: The terms O-serotype/O-serotyping have been replaced throughout the text, including the title, with O-serogroup/O-serogrouping.

Re: Spectrum00991-26R1 (Development and Validation of a High-Throughput Agglutination Assay for O-Serotyping Clinical Escherichia coli Isolates)

Dear Dr. Germie P.J.M. van den Dobbelsteen:

Your manuscript has been accepted, and I am forwarding it to the ASM production staff for publication. Your paper will first be checked to make sure all elements meet the technical requirements. ASM staff will contact you if anything needs to be revised before copyediting and production can begin. Otherwise, you will be notified when your proofs are ready to be viewed.

Data Availability: ASM policy requires that data be available to the public upon online posting of the article, so please verify all links to sequence records, if present, and make sure that each number retrieves the full record of the data. If a new accession number is not linked or a link is broken, provide production staff with the correct URL for the record. If the accession numbers for new data are not publicly accessible before the expected online posting of the article, publication may be delayed; please contact ASM production staff immediately with the expected release date.

Publication Fees: For information on publication fees and which article types have charges, please visit our website. We have partnered with Copyright Clearance Center (CCC) to collect author charges. If fees apply to your paper, you will receive a message from no-reply@copyright.com with further instructions. For questions related to paying charges through RightsLink, please contact CCC at ASM_Support@copyright.com or toll free at +1-877-622-5543. CCC makes every attempt to respond to all emails within 24 hours.

ASM Membership: Corresponding authors may join or renew ASM membership to obtain discounts on publication fees. Need to upgrade your membership level? Please contact Customer Service at Service@asmusa.org.

Embargo Policy: A press release may be issued as soon as the manuscript is posted on the Spectrum Latest Articles webpage. The corresponding author will receive an email with the subject line "ASM Journals Author Services Notification" when the article is available online.

Cover Image Submissions: If you would like to submit a potential Cover Image, please email a file and a short legend to spectrum@asmusa.org. Please note that we can only consider images that (i) the authors created or own and (ii) have not been previously published. By submitting, you agree that the image can be used under the same terms as the published article. Image File requirements: TIF/EPS, 7.5 inches wide by 8.25 inches tall (at least 2,250 pixels wide by 2,475 pixels tall), minimum 300 dpi resolution (600 dpi preferred), RGB, and no figure elements, e.g., arrows or panel labels. The legend should be a short description of the image, 1-2 sentences recommended. Please download and use this interactive template in Adobe the https://journals.asm.org/pb-assets/html/asm-cover-sizing-tool.html to ensure that your proposed cover image meets our size requirements (https://journals.asm.org/pb-assets/pdf-text-excel-files/ASM-Interactive-Sizing-Cover-Template-1715689791.pdf).

The ASM Journals program strives for constant improvement in our submission and publication process. Please tell us how we can improve your experience by taking this quick Author Survey.

Thank you for submitting your paper to Spectrum.

Sincerely,
Catherine Logue
Editor
Microbiology Spectrum

Reviewer #2 (Public repository details (Required)):

Isolate genome sequences were deposited and noted in manuscript, PRJEB97262

Reviewer #2 (Comments for the Author):

The authors have done a very nice job of responding to all reviewer concerns. This reviewer has no further concerns.
