# Supplementary material for: Development and validation of a high-throughput agglutination assay for O-serogrouping clinical Escherichia coli isolates
Source: Microbiol Spectr. 2026 Aug 21;14(10):e00991-26. doi: 10.1128/spectrum.00991-26 (PMC13689272; doi:10.1128/spectrum.00991-26)
Supplement: Supplementary file 2 [file spectrum.00991-26-s0001.docx]

**Supplemental Table S1. Agglutination Validation Panel Isolate Sources and O-Serogroup Characterization**

| **Agglutination Validation No** | **OC No.** | **Alternate Name** | **Country** | **State** | **Year** | **Patient Gender** | **Patient Age** | **Isolate Source** | **Patient Setting** | **Agglutination O-serogroup** | **Genome Sequenced** | **Molecular O-serogroup** | **O-serogroup Consensus** | **European Nucleotide Archive Accession** |
| --- | --- | --- | --- | --- | --- | --- | --- | --- | --- | --- | --- | --- | --- | --- |
| O1-1 | OC24452 | StGVXN 3736^a^ | Switzerland | - | 2012 | F | 44 | Urine | - | O1 | Yes | O1 | O1 | na |
| O1-2 | OC24685 |  | Netherlands | - | 2002 | F | 64 | Blood | - | O1 | No | - | O1 | na |
| O1-3 | OC24671 |  | Netherlands | - | 2002 | F | 81 | Blood | - | O1 | No | - | O1 | na |
| O1-4 | OC24660 |  | Netherlands | - | 2002 | M | 88 | Blood | - | O1 | No | - | O1 | na |
| O1-5 | OC24651 |  | Netherlands | - | 2012 | M | 75 | Blood | - | O1 | Yes | O1 | O1 | PRJEB97262^c^ |
| O1-6 | OC24779 |  | Switzerland | - | 2012 | F | 53 | Urine | - | O1 | Yes | O1 | O1 | na |
| O1-7 | OC24162 |  | USA | Iowa | 2011 | M | 69 | Blood | Inpatient | O1 | No | - | O1 | na |
| O1-8 | OC24199 |  | USA | Minnesota | 2011 | F | 67 | Blood | Inpatient | O1 | Yes | O1 | O1 | PRJEB97262 |
| O1-9 | OC24180 |  | USA | New Jersey | 2011 | F | 84 | Blood | Inpatient | O1 | No | - | O1 | na |
| O1-10 | OC24226 |  | USA | Georgia | 2011 | M | 62 | Blood | Inpatient | O1 | No | - | O1 | na |
| O2-1 | OC24453 | StGVXN 3220 ^a^ | - | - | - | - | - | - | - | O2 | Yes | O2 | O2 | na |
| O2-2 | OC24599 |  | Netherlands | - | 2011 | M | 68 | Blood | - | O2 | Yes | O2 | O2 | PRJEB97262 |
| O2-3 | OC24631 |  | Netherlands | - | 2012 | F | 71 | Blood | - | O2 | Yes | O2 | O2 | PRJEB97262 |
| O2-4 | OC24630 |  | Netherlands | - | 2012 | M | 67 | Blood | - | O2 | Yes | O2 | O2 | PRJEB97262 |
| O2-5 | OC24625 |  | Netherlands | - | 2012 | F | 62 | Blood | - | O2 | Yes | O2 | O2 | PRJEB97262 |
| O2-6 | OC24615 |  | Netherlands | - | 2011 | M | 68 | Blood | - | O2 | Yes | O2 | O2 | PRJEB97262 |
| O2-7 | OC24780 |  | Switzerland | - | 2012 | F | 50 | Urine | - | O2 | Yes | O2 | O2 | na |
| O2-8 | OC24230 |  | USA | Louisiana | 2011 | M | 76 | Blood | Inpatient | O2 | Yes | O2 | O2 | PRJEB97262 |
| O2-9 | OC24147 |  | USA | New York | 2011 | M | 68 | Blood | Inpatient | O2 | No | - | O2 | na |
| O2-10 | OC24333 |  | USA | Missouri | 2011 | M | 81 | Blood | Inpatient | O2 | Yes | O2 | O2 | PRJEB97262 |
| O4-1 | OC24137 |  | USA | Indiana | 2011 | F | 90 | Blood | Inpatient | O4 | Yes | O4 | O4 | PRJEB97262 |
| O4-2 | OC24499 |  | Netherlands | - | 2007 | M | 60 | Blood | - | O4 | Yes | O4 | O4 | na |
| O4-3 | OC24573 |  | Netherlands | - | 2010 | M | 62 | Blood | - | O4 | Yes | O4 | O4 | na |
| O4-4 | OC24733 |  | Netherlands | - | 2005 | F | 63 | Blood | - | O4 | Yes | O4 | O4 | na |
| O4-5 | OC24743 |  | Netherlands | - | 2005 | F | 86 | Blood | - | O4 | Yes | O4 | O4 | na |
| O4-6 | OC24750 |  | Netherlands | - | 2005 | M | 75 | Blood | - | O4 | Yes | O4 | O4 | na |
| O4-7 | OC24273 |  | USA | Colorado | 2011 | M | 78 | Blood | Inpatient | O4 | No | - | O4 | na |
| O4-8 | OC24313 |  | USA | Nevada | 2011 | F | 68 | Blood | Inpatient | O4 | Yes | O4 | O4 | PRJEB97262 |
| O4-9 | OC24318 |  | USA | Nebraska | 2011 | F | 69 | Blood | Inpatient | O4 | Yes | O4 | O4 | PRJEB97262 |
| O4-10 | OC24334 |  | USA | Washington State | 2011 | M | 85 | Blood | Inpatient | O4 | Yes | O4 | O4 | PRJEB97262 |
| O6-1 | OC24454 | StGVXN 3488^a^; CFT073 | - | Maryland | na | F | na | Blood and Urine | - | O6 | Yes | O6 | O6 | na |
| O6-2 | OC24580 |  | Netherlands | - | 2010 | F | 62 | Blood | - | O6 | Yes | O6 | O6 | na |
| O6-3 | OC24575 |  | Netherlands | - | 2010 | M | 76 | Blood | - | O6 | No | - | O6 | na |
| O6-4 | OC24549 |  | Netherlands | - | 2009 | M | 81 | Blood | - | O6 | No | - | O6 | na |
| O6-5 | OC24567 |  | Netherlands | - | 2010 | F | 77 | Blood | - | O6 | No | - | O6 | na |
| O6-6 | OC24572 |  | Netherlands | - | 2010 | - | 72 | Blood | - | O6 | No | - | O6 | na |
| O6-7 | OC24781 | stGVXN3561^a^ | - | - | - | - | - | - | - | O6 | Yes | O6 | O6 | na |
| O6-8 | OC24149 |  | USA | New York | 2011 | F | 74 | Blood | Inpatient | O6 | Yes | O6 | O6 | PRJEB97262 |
| O6-9 | OC24204 |  | USA | California | 2011 | M | 90 | Blood | Inpatient | O6 | No | - | O6 | na |
| O6-10 | OC9488 | ATCC 700928^b^; CFT073 | na | Maryland | na | F | na | Blood and Urine | na | O6 | na | O6 | O6 | na |
| O15-1 | OC24179 |  | USA | New Jersey | 2011 | F | 83 | Blood | Inpatient | O15 | Yes | O15 | O15 | PRJEB97262 |
| O15-2 | OC24484 |  | Netherlands | - | 2007 | M | 72 | Blood | - | O15 | No | - | O15 | na |
| O15-3 | OC24511 |  | Netherlands | - | 2008 | M | 62 | Blood | - | O15 | No | - | O15 | na |
| O15-4 | OC24571 |  | Netherlands | - | 2010 | M | 76 | Blood | - | O15 | No | - | O15 | na |
| O15-5 | OC24576 |  | Netherlands | - | 2010 | M | 70 | Blood | - | O15 | No | - | O15 | na |
| O15-6 | OC24601 |  | Netherlands | - | 2011 | M | 67 | Blood | - | O15 | Yes | O15 | O15 | PRJEB97262 |
| O15-7 | OC24243 |  | USA | Colorado | 2011 | M | 76 | Blood | Inpatient | O15 | Yes | O15 | O15 | PRJEB97262 |
| O15-8 | OC24224 |  | USA | Minnesota | 2011 | F | 86 | Blood | Inpatient | O15 | Yes | O15 | O15 | PRJEB97262 |
| O15-9 | OC24209 |  | USA | New Jersey | 2011 | F | 85 | Blood | Inpatient | O15 | Yes | O15 | O15 | PRJEB97262 |
| O15-10 | OC24352 |  | USA | Connecticut | 2011 | F | 76 | Blood | Inpatient | O15 | Yes | O15 | O15 | PRJEB97262 |
| O16-1 | OC24208 |  | USA | Indiana | 2011 | M | 61 | Blood | Inpatient | O16 | Yes | O16 | O16 | PRJEB97262 |
| O16-2 | OC24221 |  | USA | South Carolina | 2011 | M | 70 | Blood | Inpatient | O16 | Yes | O16 | O16 | PRJEB97262 |
| O16-3 | OC24270 |  | USA | New Jersey | 2011 | F | 63 | Blood | Inpatient | O16 | Yes | O16 | O16 | PRJEB97262 |
| O16-4 | OC24350 |  | USA | Nevada | 2011 | M | 72 | Blood | Inpatient | O16 | Yes | O16 | O16 | PRJEB97262 |
| O16-5 | OC24378 |  | USA | West Virginia | 2011 | F | 61 | Blood | Inpatient | O16 | Yes | O16 | O16 | PRJEB97262 |
| O16-6 | OC24308 |  | USA | New York | 2011 | F | 75 | Blood | Inpatient | O16 | Yes | O16 | O16 | PRJEB97262 |
| O16-7 | OC24254 |  | USA | Wisconsin | 2011 | F | 83 | Blood | Inpatient | O16 | Yes | O16 | O16 | PRJEB97262 |
| O16-8 | OC24312 |  | USA | New York | 2011 | F | 86 | Blood | Inpatient | O16 | Yes | O16 | O16 | PRJEB97262 |
| O16-9 | OC24367 |  | USA | Missouri | 2011 | M | 62 | Blood | Inpatient | O16 | Yes | O16 | O16 | PRJEB97262 |
| O16-10 | OC24556 |  | Netherlands | - | 2009 | M | 85 | Blood | - | O16 | Yes | O16 | O16 | na |
| O18-1 | OC24271 |  | USA | New Jersey | 2011 | M | 70 | Blood | Inpatient | O18 | Yes | O18 | O18 | PRJEB97262 |
| O18-2 | OC24493 |  | Netherlands | - | 2007 | M | 74 | Blood | - | O18 | No | - | O18 | na |
| O18-3 | OC24498 |  | Netherlands | - | 2007 | F | 69 | Blood | - | O18 | No | - | O18 | na |
| O18-4 | OC24548 |  | Netherlands | - | 2009 | F | 61 | Blood | - | O18 | No | - | O18 | na |
| O18-5 | OC24569 |  | Netherlands | - | 2010 | M | 63 | Blood | - | O18 | No | - | O18 | na |
| O18-6 | OC24187 |  | USA | Georgia | 2011 | M | 76 | Blood | Inpatient | O18 | Yes | O18 | O18 | PRJEB97262 |
| O18-7 | OC24247 |  | USA | Indiana | 2011 | M | 66 | Blood | Inpatient | O18 | Yes | O18 | O18 | PRJEB97262 |
| O18-8 | OC24203 |  | USA | California | 2011 | M | 83 | Blood | Inpatient | O18 | Yes | O18 | O18 | PRJEB97262 |
| O18-9 | OC24255 |  | USA | Wisconsin | 2011 | F | 73 | Blood | Outpatient | O18 | Yes | O18 | O18 | PRJEB97262 |
| O18-10 | OC24590 |  | Netherlands | - | 2010 | F | 78 | Blood | - | O18 | No | - | O18 | na |
| O25-1 | OC24455 |  | Switzerland | - | 2012 | M | 63 | Urine | - | O25 | Yes | O25 | O25 | na |
| O25-2 | OC24531 |  | Netherlands | - | 2009 | M | 63 | Blood | - | O25 | No | - | O25 | na |
| O25-3 | OC24524 |  | Netherlands | - | 2008 | M | 66 | Blood | - | O25 | No | - | O25 | na |
| O25-4 | OC24521 |  | Netherlands | - | 2008 | F | 62 | Blood | - | O25 | No | - | O25 | na |
| O25-5 | OC24519 |  | Netherlands | - | 2008 | M | 65 | Blood | - | O25 | No | - | O25 | na |
| O25-6 | OC24504 |  | Netherlands | - | 2008 | M | 71 | Blood | - | O25 | No | - | O25 | na |
| O25-7 | OC24782 |  | Switzerland | - | 2012 | F | 57 | Urine | - | O25 | Yes | O25 | O25 | na |
| O25-8 | OC24161 |  | USA | Iowa | 2011 | M | 82 | Blood | Inpatient | O25 | No | - | O25 | na |
| O25-9 | OC24154 |  | USA | Georgia | 2011 | F | 68 | Blood | Inpatient | O25 | No | - | O25 | na |
| O25-10 | OC24143 |  | USA | Michigan | 2011 | M | 62 | Blood | Inpatient | O25 | No | - | O25 | na |
| O75-1 | OC24184 |  | USA | Wisconsin | 2011 | M | 68 | Blood | Inpatient | O75 | Yes | O75 | O75 | PRJEB97262 |
| O75-2 | OC24507 |  | Netherlands | - | 2008 | F | 82 | Blood | - | O75 | Yes | O75 | O75 | na |
| O75-3 | OC24558 |  | Netherlands | - | 2009 | F | 70 | Blood | - | O75 | Yes | O75 | O75 | na |
| O75-4 | OC24713 |  | Netherlands | - | 2003 | M | 82 | Blood | - | O75 | No | - | O75 | na |
| O75-5 | OC24729 |  | Netherlands | - | 2004 | F | 70 | Blood | - | O75 | Yes | O75 | O75 | na |
| O75-6 | OC24761 |  | Netherlands | - | 2006 | M | 80 | Blood | - | O75 | Yes | O75 | O75 | na |
| O75-7 | OC24244 |  | USA | Minnesota | 2011 | M | 77 | Blood | Inpatient | O75 | Yes | O75 | O75 | PRJEB97262 |
| O75-8 | OC24181 |  | USA | Wisconsin | 2011 | M | 67 | Blood | Inpatient | O75 | Yes | O75 | O75 | PRJEB97262 |
| O75-9 | OC24283 |  | USA | Wisconsin | 2011 | F | 77 | Blood | Outpatient | O75 | Yes | O75 | O75 | na |
| O75-10 | OC24321 |  | USA | Nebraska | 2011 | M | 69 | Blood | Inpatient | O75 | Yes | O75 | O75 | PRJEB97262 |
| O21 | OC24296 |  | USA | Wisconsin | 2011 | M | 71 | Blood | Inpatient | O21 | Yes | O21 | O21 | PRJEB97262 |
| O22 | OC24369 |  | USA | Missouri | 2011 | M | 73 | Blood | Inpatient | O22 | Yes | O22 | O22 | PRJEB97262 |
| O44 | OC24213 |  | USA | Florida | 2011 | F | 76 | Blood | Inpatient | O44 | Yes | O17_O44_O73_O77_O106 | O17_O44_O73_O77_O106 | PRJEB97262 |
| O88 | OC24241 |  | USA | Georgia | 2011 | F | 65 | Blood | Inpatient | O88 | Yes | O88 | O88 | PRJEB97262 |
| O153 | OC24206 |  | USA | California | 2011 | F | 83 | Blood | Inpatient | O153 | Yes | O153 | O153 | PRJEB97262 |

^a^ Strains acquired from GlycoVaxyn

^b^ Strain acquired directly from ATCC

^c^ European Nucleotide Archive Accession [PRJEB97262](https://www.ebi.ac.uk/ena/browser/view/PRJEB97262)

**Supplementary Protocol: High-Throughput Agglutination Assay for O-Serogroup Characterization of *Escherichia coli* Isolates**

# 1. **Overview**

This protocol describes a high-throughput agglutination assay for serotyping *Escherichia coli* isolates using heat-treated bacterial suspensions and commercially available O-serogroup antisera. Agglutination reactions are quantified using automated image analysis based on Total Intensity of Spots per Well (TIOSPW) measurements generated by a CTL ImmunoSpot® Analyzer.

# 2. **Materials and Reagents**

## Reagents

- Formaldehyde solution (36–38%)
- Sodium chloride (NaCl)
- 10X phosphate-buffered saline (PBS)
- Sterile cell culture-grade water
- 0.85% saline solution, pH 7.4
- Veal infusion agar
- Yeast extract
- Dextrose
- High-titer *E. coli* antisera specific for O1, O2, O4, O6, O15, O16, O18ac, O25, and O75 serogroups

## Consumables

- Six-well tissue culture plates
- Vented aluminum 96-well glass tube kit
- Clear flat-bottom 96-well microplates
- Round-bottom 96-well microtiter plates
- Sealing mats and lids
- Wide-orifice pipette tips

## Equipment

- Autoclave
- Biosafety cabinet
- Incubator capable of maintaining 35–37°C
- Incubator capable of maintaining 50–52°C
- Water bath capable of maintaining 95°C
- Microplate reader capable of OD_600_ measurements
- CTL ImmunoSpot® Analyzer or equivalent imaging system
- Multichannel pipettes and/or automated liquid handler

# 3. **Preparation of Reagents and Media**

## 3.1 Formal Saline Solution (FSS)

Prepare formal saline solution containing a final concentration of 0.216% formaldehyde and 0.55% NaCl.

For 1 L of FSS:

1. Dissolve 5.5 g NaCl in approximately 994 mL distilled water.
2. Add sufficient formaldehyde stock solution to achieve a final concentration of 0.216% formaldehyde. The volume added should be adjusted according to the actual concentration stated on the certificate of analysis.
3. Adjust the final volume to 1 L with distilled water.
4. Mix thoroughly and store at room temperature.

## 3.2 Preparation of 1X PBS

Prepare 1X PBS by diluting 10X PBS tenfold with sterile water.

## 3.3 Veal Infusion/Yeast Extract Agar (VIA)

For 500 mL medium:

- 20 g veal infusion agar
- 1 g yeast extract
- 0.5 g dextrose
- 500 mL distilled water

Mix until dissolved and autoclave at 121°C for 15 min. Cool to approximately 60°C and dispense approximately 7 mL into each well of a six-well plate. Allow agar to solidify and store at 4°C until use.

# 4. **Growth of *E. coli* Cultures**

1. Streak frozen *E. coli* isolates and serogroup control strains onto VIA plates to obtain isolated colonies.
2. Incubate plates at 35–37°C for 16–20 h.
3. Use freshly isolated colonies for preparation of bacterial suspensions.

# 5. **Preparation and Standardization of Bacterial Suspensions**

1. Dispense 2 mL FSS into each well of a vented aluminum 96-well glass tube block
2. Using wide-orifice pipette tips or sterile loops, transfer isolated colonies into FSS.
3. Dislodge colonies by repeated pipetting.
4. Thoroughly resuspend bacterial growth until a homogeneous suspension is obtained.
5. Transfer 200 μL of each suspension into a clear flat-bottom 96-well plate.
6. Measure OD_600_ using a microplate reader.

## 5.1 Optical Density Adjustment

Adjust all suspensions to an OD_600_ between 0.60 and 1.24.

- If OD_600_ is below 0.60, add additional colony biomass.
- If OD_600_ is above 1.24, dilute with FSS.

Repeat measurements as needed until all suspensions fall within the acceptable range.

# 6. **Heat Treatment of Bacterial Suspensions**

1. Seal the 96-well block with a sealing mat and lid.
2. Incubate in a 95°C water bath for 2 h.
3. Remove from the water bath and allow suspensions to cool at room temperature for at least 1 h.

The cooling period allows sedimentation of cellular debris and should be maintained before assay setup.

# 7. **Preparation of Antisera**

High-titer O-antisera should be diluted immediately before use according to the manufacturer’s instructions using 0.85% saline solution (pH 7.4).

The following antisera were evaluated:

- O1
- O2
- O4
- O6
- O15
- O16
- O18ac
- O25
- O75

# 8. **Agglutination Assay**

## 8.1 Negative-Control Plate

1. Add 50 μL of 1X PBS to each well of a round-bottom 96-well microtiter plate.
2. Add 50 μL of cooled bacterial suspension to each well.
3. Avoid disturbing sedimented debris during transfer

## 8.2 Antisera Plates

1. Prepare one round-bottom 96-well plate for each O-antiserum.
2. Dispense 50 μL of diluted O-antiserum into each well.
3. Add 50 μL of cooled bacterial suspension to each corresponding well.

The final reaction volume is 100 μL per well.

## 8.3 Positive Controls

Reference strains representing each target O-serogroup should be included in each assay run. Positive-control strains should produce positive reactions with homologous antisera and negative reactions with heterologous antisera.

# 9. **Incubation**

1. Cover plates with lids.
2. Place plates in sealed bags or containers to minimize evaporation.
3. Incubate at 50–52°C for 16–20 h in a humidified incubator.

Humidity may be maintained by placing a reservoir of water inside the incubator.

# 10. **Plate Reading and Data Acquisition**

1. Remove plates from the incubator.
2. Allow plates to equilibrate to room temperature for 20–30 min.
3. Do not agitate or mix plates prior to reading.
4. Visually inspect wells and acquire images using a CTL ImmunoSpot Analyzer.

Agglutination reactions are quantified using the software-generated parameter Total Intensity of Spots per Well (TIOSPW), which measures the intensity of the button formed at the bottom of each well.

# 11. **Interpretation of Results**

## 11.1 Visual Interpretation

Positive agglutination reactions appear as cloudy-to-clear wells lacking a compact central button.

Negative reactions produce a distinct compact button centered at the bottom of the well.

## 11.2 Automated Interpretation

For the assay described in this study, a validation-derived cutoff of 11.65 TIOSPW was established and used to classify reactions.

- Positive reaction: TIOSPW < 11.65
- Negative reaction: TIOSPW ≥ 11.65

The cutoff value of 11.65 was established during assay validation using the specific instrument configuration, image acquisition settings, bacterial suspension preparation procedure, and OD_600_ range described herein.

Laboratories implementing this method should perform local validation studies to confirm assay performance and determine an appropriate classification threshold under their own operating conditions. Variations in instrument calibration, image analysis settings, bacterial biomass, culture density, or other assay parameters may influence the optimal TIOSPW cutoff.

# 12. **Acceptance Criteria**

## 12.1 Bacterial Suspensions

Bacterial suspensions must have an OD_600_ between 0.60 and 1.24 prior to heat treatment.

## 12.2 Negative-Control Wells

For each isolate, the corresponding PBS control well must:

- Produce a compact central button, and
- Generate a TIOSPW value consistent with a negative reaction.

If the negative control fails, the isolate should be retested on the PBS plate and all antisera plates.

## 12.3 Positive-Control Strains

### Homologous Reactions:

Positive-control strains must generate positive reactions with their corresponding antisera.

### Heterologous Reactions:

Positive-control strains must generate negative reactions with non-corresponding antisera.

Failure of a positive control invalidates the corresponding antisera plate and requires repeat testing.

# 13. **Critical Notes**

1. Use freshly isolated colonies from 16–20 h cultures.
2. Ensure complete resuspension of colonies before OD_600_ measurement.
3. Maintain OD_600_ within the validated range of 0.60–1.24.
4. Avoid disturbing sedimented debris when transferring heat-treated suspensions.
5. Do not agitate assay plates before image acquisition.
6. Include appropriate homologous and heterologous positive-control strains in every assay run.
7. Perform antiserum dilutions on the day of use according to manufacturer recommendations.
